# Supplementary material for: The interactome of intact mitochondria by cross-linking mass spectrometry provides evidence for coexisting respiratory supercomplexes
Source: Mol Cell Proteomics. 2017 Dec 8;17(2):216–32. doi: 10.1074/mcp.RA117.000470 (PMC5795388; doi:10.1074/mcp.RA117.000470)
Supplement: Supplemental Data [file supp_RA117.000470_133922_0_supp_23978_fzffwf.zip › spectra_annotation/mito_spectra_annotation/157-1-4-1-3-1.pdf]

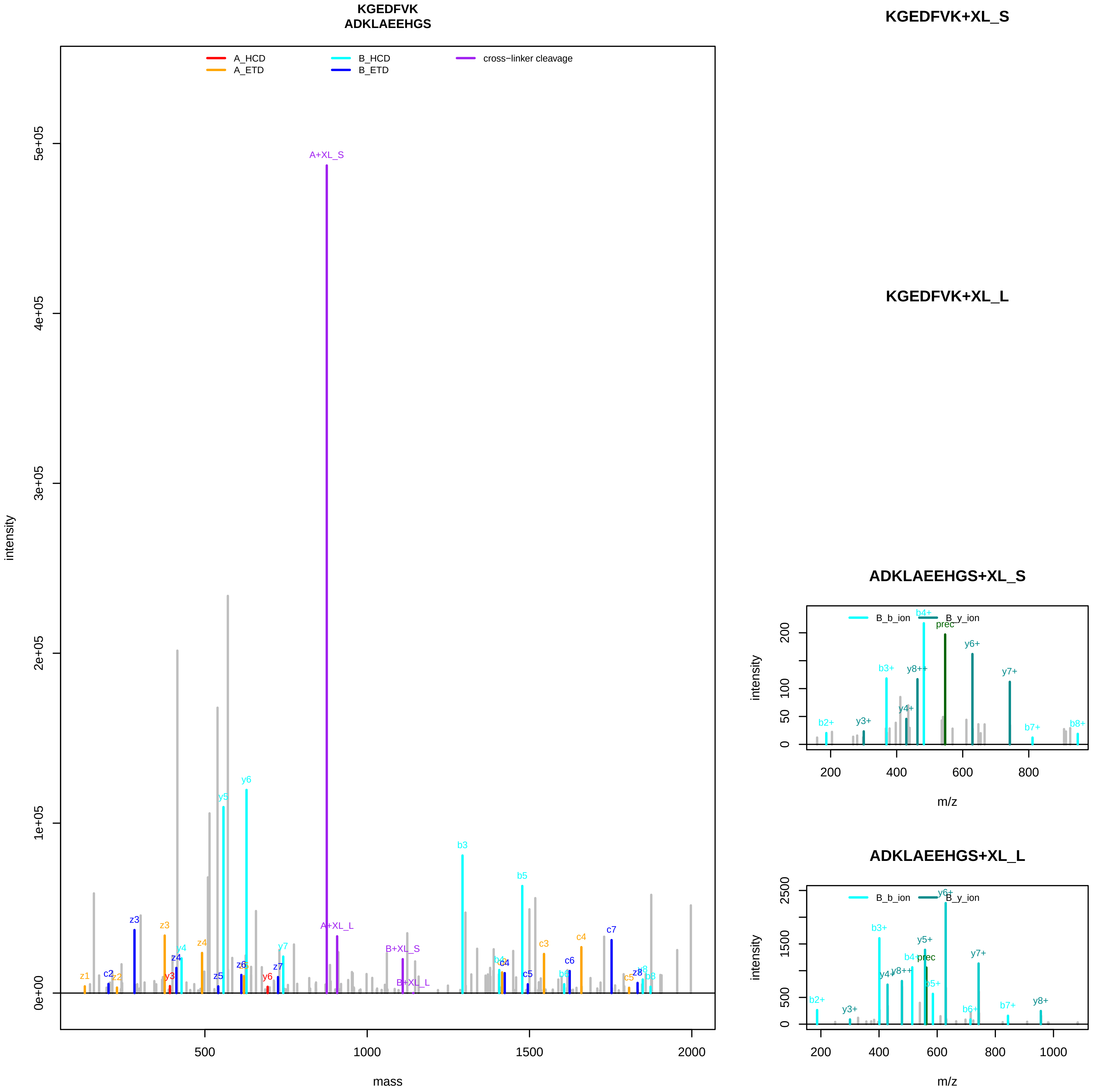
KGEDFVK
ADKLAEEHGS
A_HCD
A_ETD
B_HCD
B_ETD
cross−linker cleavage
intensity
mass
0e+00
1e+05
2e+05
3e+05
4e+05
5e+05
500
1000
1500
2000
A+XL_S
A+XL_L
B+XL_S
B+XL_L
z1 c2 z2 z3 z3 y3 z4 y4 z4 z5 y5 z6 y6 y6 y7 z7
b3 b5 b4 c4 c5 c3 b6 c6 c4 c7 c5 z8 y8 b8
KGEDFVK+XL_S
KGEDFVK+XL_L
ADKLAEEHGS+XL_S
B_b_ion
B_y_ion
prec
b2+ y3+ b3+ y4+ y8++ b4+ y6+ y7+ b7+ b8+
m/z
ADKLAEEHGS+XL_L
b2+ y3+ b3+ y4+ y8++ b4+ y5+ b5+ y6+ prec y7+ b6+ b7+ y8+
200
400
600
800
1000
0
50
100
200
500
1500
2500
